# Editorial: Interdisciplinary approaches to complex systems: highlights from FRCCS 2023/24

**DOI:** 10.3389/fdata.2025.1666305

**Published:** 2025-08-12

**Authors:** Roberto Interdonato, Hocine Cherifi

**Affiliations:** ^1^CIRAD, UMR TETIS, Montpellier, France; ^2^Inria, University of Montpellier, Montpellier, France; ^3^Laboratoire Interdisciplinaire Carnot de Bourgogne, Université Bourgogne Europe, Dijon, France

**Keywords:** complex system, complex network, spatial complexity, urban network, large language model, physics-guided deep learning, sustainable development goals

The study of complex systems has emerged as one of the most compelling and transformative fields in contemporary science, offering profound insights into the fundamental nature of organization, emergence, and adaptation across scales from molecular networks to global ecosystems, from neural circuits to social institutions. This research topic presents a curated selection of research contributions that exemplify the rich interdisciplinary dialogue fostered by the France's International Conference on Complex Systems (FRCCS) through its third and fourth editions, held in Le Havre (2023) and Montpellier (2024). The contributions in this Research Topic exemplify the evolution of complexity science toward data-driven, computationally sophisticated, and socially relevant research. The selected articles demonstrate how traditional complexity science foundations are being enhanced by cutting-edge computational methods and applied to pressing contemporary challenges.

For instance, the exploration of physics-guided machine learning approaches for predicting chaotic system dynamics illustrates how domain knowledge can enhance data-driven methods, offering new pathways for understanding and forecasting complex behaviors. Feng et al. introduce a Physics-Guided Learning (PGL) approach that combines empirical observations with fundamental physical principles to improve prediction accuracy in chaotic systems over extended time horizons. Rather than relying solely on data patterns or physical equations, the method creates a hybrid framework that integrates three complementary components: a Data-Driven Component (for extracting patterns and relationships from historical observations), a Physics-Guided Component (that incorporates physical laws to guide and constrain the learning process) and a Nonlinear Learning Component to combines insights from both data and physics. Evaluation on six dynamical systems, each exhibiting unique chaotic behaviors, show the significance of the proposed approach. Always at the edge between complex systems and AI, the evaluation of fine-tuning vs. prompting strategies for knowledge graph construction using large language models demonstrates how modern AI architectures can be leveraged to extract and represent complex relationships from unstructured data. Ghanem and Cruz focus on how to exploit Large Language Models (LLMs) for a Text-to-Knowledge Graph (T2KG) construction task. By testing different strategies (Zero-Shot Prompting, Few-Shot Prompting, and Fine-Tuning) on three LLMs (Llama2, Mistral, and Starling) they show the potential of these techniques in the context of Knowledge Graph construction. While, also through the introduction of nuanced evaluation metrics, the authors show how Fine-Tuning tends to outperform other strategies, they also emphasize how this approach can limit generalization capablities of the resulting model.

*Urban systems and spatial complexity* emerge as a central theme, reflecting the growing recognition of cities as archetypal complex systems. Bogomolov et al. develop a methodological framework for urban delineation using passive mobile phone data to analyze commute patterns in the city of Brno (Czech Republic). The authors focus on the analysis of bidirectional commute flows, by examining movement patterns in urban and suburban networks, creating a comprehensive view of mobility that spans city boundaries. This allows to identify mobility patterns and communities (based on clustering techniques) that are based on actual movements, rather than on road networks or administrative boundaries. The results show how this framework can be helpful for urban planners, since it allows to assess the centrality of certain zones regardless of their geographical location (e.g., areas with shopping centers being considered central for their economic activity, even if geographically peripheral). Complementing this, the analysis of city composition and accessibility statistics in and around Paris provides concrete insights into how urban complexity manifests in terms of spatial organization and resource distribution. Thaury et al. examine whether Paris truly functions as a “15-minute city” where residents can access essential amenities within a 15-min walk or bike ride, using a combination of open-source mapping data and official socio-economic statistics. The authors developed a comprehensive accessibility framework that goes beyond simply counting nearby amenities to measure actual accessibility based on supply, demand, and distance factors. The analysis reveals a stark dichotomy within Paris itself, with a highly accessible and well-equipped center contrasting sharply with less-served peripheral neighborhoods. A significant finding concerns socio-economic inequalities in accessibility. Poorer neighborhoods, predominantly located on the city's edges, have substantially less access to amenities. The key takeaway message of the work is that, starting from the observation that Paris fails to meet the 15-minute city ideal due to persistent center-periphery inequalities, successful implementation of this concept requires addressing existing socio-economic disparities and adopting a metropolitan-scale perspective rather than focusing solely on the central municipality.

*Technological integrity and security* in complex systems has gained paramount importance in our interconnected world. Rani et al. address the growing threat of deepfake technology, which has reached alarming levels with ~71% of people falling victim to fake video-based blackmail and manipulation. The researchers developed a hybrid architecture that combines transformer and Linformer models (i.e., transformer variants optimized for computational efficiency) to create a more efficient and accurate deepfake detection system. The research demonstrates that the use of larger patch sizes improve performance by enabling better capture of fine-grained features and spatial details, which enhances the model's ability to distinguish between authentic and manipulated content. This approach addresses the critical need for detection systems that are both highly accurate and computationally feasible for real-world deployment.

*Sustainable development and knowledge systems* represent an increasingly crucial application domain. Paletta et al. introduce a methodological framework for visualizing the alignment between complex research systems and the Sustainable Development Goals (SDGs). The authors use a French research institute, namely CIRAD (French Agricultural Research Centre for International Development) as case in point, showing how the proposed visualization allows to explore thematic priorities and institutional collaborations. The integration of complex systems theory and network analysis enhances understanding of SDG interlinkages and provides actionable insights for strategic decision-making in research governance.

The emphasis on research with high societal impact has been a distinguishing feature of FRCCS. The contributions in this Research Topic demonstrate how complexity science is moving beyond academic discourse to inform policy, guide intervention strategies, and shape technological development. This applied dimension does not diminish the theoretical rigor of the work but rather enhances its relevance and validates its insights.

